# Focused Ultrasound Neuromodulation and the Confounds of Intracellular Electrophysiological Investigation

**DOI:** 10.1523/ENEURO.0213-20.2020

**Published:** 2020-08-20

**Authors:** Morgan N. Collins, Karen A. Mesce

**Affiliations:** 1 Graduate Program in Neuroscience, University of Minnesota, St. Paul, MN 55108; 2 Departments of Entomology and Neuroscience, University of Minnesota, St. Paul, MN 55108

**Keywords:** cavitation, electrode resonance, *Hirudo verbana*, intracellular recording, leak currents, ultrasound neuromodulation

## Abstract

Focused ultrasound (US) can modulate neuronal activity noninvasively with high spatial specificity. In intact nervous systems, however, efforts to determine its enigmatic mode of efficacy have been confounded by the indirect effects of US on mechanosensitive sensory cells and the inability to target equivalent populations of cells with precision across preparations. Single-cell approaches, either via cultured mammalian neurons or tractable invertebrate neural systems, hold great promise for elucidating the cellular mechanisms underlying the actions of US. Here, we present evidence from the medicinal leech, *Hirudo verbana*, that researchers should apply caution when using US in conjunction with single-cell electrophysiological recording techniques, including sharp-electrode intracellular recording. Although we found that US could elicit depolarization of the resting membrane potential of single neurons, a finding with precedent, we determined that this effect and others could be reliably mimicked via subtle manual displacement of the recording electrode. Because focused US is known to induce resonance of recording electrodes, we aimed to determine how similarly US-induced depolarizations matched those produced by micro movements of a sharp glass electrode, a phenomenon we believe can account for purported depolarizations measured in this manner. Furthermore, we show that when clonally related homologous neurons, which are essentially isopotential, are impaled before the application of focused US, they show a statistically significant change in their membrane potential as compared with the homologous cells that received US with no initial impalement. Future investigations into US’s cellular effects should attempt to control for potential electrode resonance or use alternative recording strategies.

## Significance Statement

Interest in focused ultrasound (US) neuromodulation has soared in recent years, yet researchers have yet to agree on whether US excites or inhibits neuronal activity, or what mechanisms underly these effects. Basic investigations have attempted to clarify how US affects neuronal membrane properties to understand how it alters firing rates. Several groups have linked US-induced excitation to depolarization of the resting membrane potential, as measured with intracellular sharp electrodes or membrane patch methods. Here, we replicate this depolarization while recording with intracellular sharp electrodes, but find that the depolarizing effects of US can be replicated by small displacements of the recording electrode. We conclude that intracellular electrophysiological investigations of US’s neuromodulatory effects are susceptible to artifacts introduced via electrode resonance.

## Introduction

Focused ultrasound (US) is currently under investigation as a promising noninvasive neuromodulation technology. Reports of the effects of US on nervous tissue date back 100 years (Harvey, 1929). Recently, the pace of US neuromodulation research has accelerated as other neuromodulatory technologies (e.g., those using implantable devices) have proven to be therapeutic for the treatment of an ever-increasing array of neurologic disorders. Uniquely among noninvasive technologies, US has the ability to deliver energy noninvasively to deep brain structures with high spatial specificity (Hynynen and Clement, 2007; Ai et al., 2016).

Despite evidence that US modulates neuronal activity in a wide range of animal systems, including humans (Legon et al., 2014, 2018), inconsistencies in reported outcomes persist with respect to the direction of its effects. Researchers have reported both US-induced neuronal excitation (Tyler et al., 2008; Tufail et al., 2010; Yoo et al., 2011; Kim et al., 2012, 2014; Downs et al., 2018) and inhibition (Fry et al., 1958; Rinaldi et al., 1991; Min et al., 2011; Legon et al., 2014, 2018; Kim et al., 2015). Furthermore, underlying mechanisms to account for the neuronal excitatory and inhibitory actions of US have been ascribed to being thermal (Lele, 1963; Colucci et al., 2009; Darrow et al., 2019), mechanical (direct or via US-induced cavitation; Plaksin et al., 2014; Wright et al., 2017; Kubanek et al., 2018; Menz et al., 2019), or a combination of the two (Bachtold et al., 1998). Efforts to elucidate how US modulates neural activity have been confounded by US activation of mechanosensory structures, including auditory hair cells (Guo et al., 2018; Sato et al., 2018). To circumvent these and other complicating factors, we and other groups have examined how US influences neurons on a foundational level in tractable invertebrate systems (Wright et al., 2015, 2017; Yoo et al., 2017; Kubanek et al., 2018; Dedola et al., 2020), mammalian cell culture (Muratore et al., 2009; Qiu et al., 2019), or slice (Rinaldi et al., 1991; Bachtold et al., 1998; Tyler et al., 2008; Prieto et al., 2018).

Recently, we obtained evidence to support the idea that the direct effects of US on nerves at low intensities are largely inhibitory (Mesce and Newhoff, 2020; M. N. Collins, W. Legon, and K. A. Mesce, unpublished observations). We obtained these results by studying a synaptically-isolated identified motoneuron in the well-studied medicinal leech, *Hirudo verbana*. This work stands in contrast to some other single-cell reports whereby US was found to induce neuronal excitation via depolarization of the resting membrane potential (Tyler et al., 2008; Lin et al., 2019; Dedola et al., 2020). Because we used extracellular suction electrodes versus intracellular or patch electrodes to record action potentials from the axons of our identified neuron, we considered whether different recording methodologies might contribute to a phenomenon of excitation versus inhibition.

Here, we examined the effects of US on the resting membrane potentials of identified leech neurons, and asked whether the actions of US could be influenced by the impalement of a sharp-glass electrode. As in vertebrate neurons, the rising and falling phases of its action potential are mediated by voltage-gated sodium and potassium channels, respectively (Kleinhaus, 1976; Kleinhaus and Prichard, 1976). This is important to note, as these channels have been implicated as actuators of US-induced neuromodulation, yet are not present in all animal models under investigation with US (e.g., *Caenorhabditis elegans* lacks voltage-gated sodium channels).

As our primary target, we chose the Retzius neuron, a serotonergic bilaterally-paired cell located on the ventral surface of all 21 segmental ganglia. This cell has been extensively studied since its discovery in 1891 (Carretta, 1988). Its large soma (50–80 μm in diameter) has enabled its rapid identification and subsequent impalement during intracellular recording experiments. The two Retzius neurons per segmental ganglion are electrotonically coupled and nearly isopotential (Hagiwara and Morita, 1962; Eckert, 1963). To compare our findings with a recent intracellular investigation of US on leech nociceptive (N) cells (Dedola et al., 2020), we performed additional experiments on this cell type.

Specifically, we studied whether physical microadjustments of the intracellular electrode could mimic the depolarized state and related action potential parameters induced by US. We found that US-induced changes, including depolarization of the resting membrane potential, an increase in spike frequency, and attenuation of spike amplitude could be mimicked by brief, manual electrode displacements. Because of known US-induced electrode resonance, the rapid depolarization of cells found to occur in neurons in response to US application during intracellular recording may be artifactual, as we have found here.

## Materials and Methods

### Animal preparation

**Figure 1.**
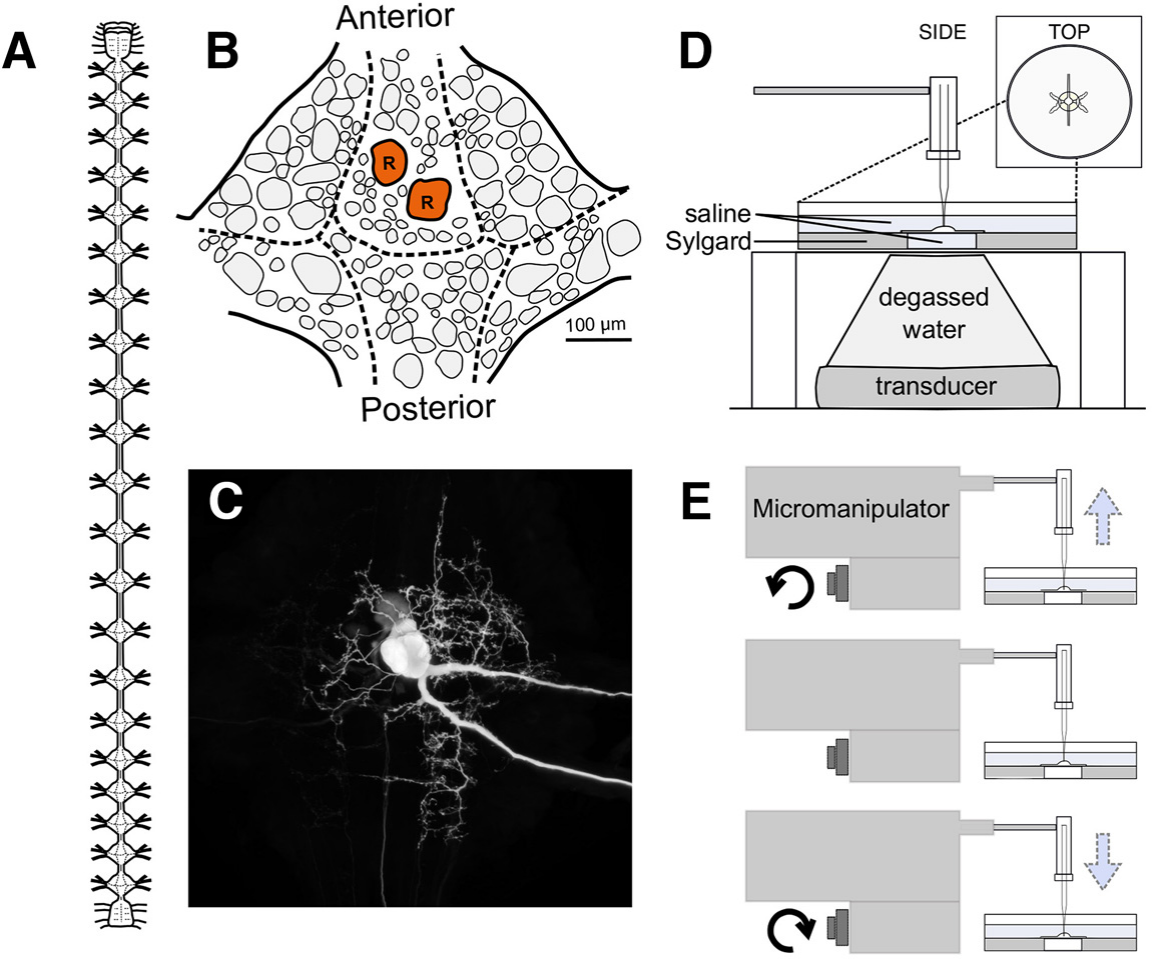
The medicinal leech and experimental design. ***A***, Diagram of the central nervous system of the leech, characterized by a ventral nerve cord interspersed with 21 segmental ganglia descending from a compound cephalic ganglion. ***B***, Schematic of the placement of neuronal somata on the ventral surface of a single ganglion. The bilateral Retzius cells are colored red and labeled R. ***C***, Neurobiotin fill of a Retzius cell showing its soma, neurites, and axons (a faintly labeled contralateral soma is present because of electrical coupling of the two cells). ***D***, US paradigm demonstrating the positioning of the transducer, intracellular electrode, and ganglion preparation. ***E***, Side view of the electrode displacement paradigm demonstrating the movement of the recording electrode.

We examined the effects of US and manual electrode displacement on Retzius neurons from the medicinal leech, *H. verbana*. Retzius cells are present bilaterally in each of the leech’s 21 segmental ganglia; a diagram of the leech nervous system and a single ganglion are shown in Figure 1*A*,*B*. Retzius cells can be readily identified because of their large size and firing properties, enabling rapid entry and reentry of the same cell. The resting membrane potential is typically −30 to −50 mV, and spikes are 20–50 mV in amplitude (Hagiwara and Morita, 1962; Eckert, 1963). The cell’s soma and neurites are visible in a Neurobiotin cell fill in Figure 1*C*.

We obtained hermaphroditic adult leeches from Niagara Medical Leeches; they were housed at room temperature (22–24°C) in a large tank filled with pond water and anaesthetized on ice before dissection. Single leech ganglia were pinned ventral side up in a Petri dish lined with 2-mm-thick SYLGARD (Dow Corning) and filled with leech saline (115 mm NaCl, 4.0 mm KCl, 1.8 mm CaCl_2_, 1.5 mm MgCl_2_, 10.0 mm glucose, and 10.0 mm Trizma preset crystals, all from Sigma-Aldrich; recipe adapted from Nicholls and Baylor, 1968). A 5-mm diameter circle of SYLGARD directly beneath the ganglion was removed, and the hole in the dish was sealed with a thin layer of latex.

### Intracellular recording

The somata of Retzius neurons were impaled with sharp electrodes made from borosilicate glass (1 mm in outer diameter, 0.75 mm in inner diameter) pulled to resistances of 25–40 MΩ on a micropipette puller (P-87, Sutter Instrument Co); electrodes were filled with 2 m potassium acetate and 20 mm KCl (Cymbalyuk et al., 2002). Recordings were amplified (IX2-700 dual intracellular preamp, Dagan Corp.), digitized (Axon CNS Digidata 1440A, Molecular Devices), and bridge balanced. Data were acquired with pClamp software (Molecular Devices) and imported into MATLAB (R2018b, MathWorks) for analysis.

The US transducer (Sonic Concepts H102-MR) was placed beneath the preparation (Fig. 1*D*, schematic). The degassed, deionized water-filled focusing cone was sealed to the latex-covered dish opening with a drop of water, ensuring continuous transmission of energy from the transducer to the ganglion.

### Neurobiotin cell filling

The Retzius cell fill displayed in Figure 1*C* was filled by iontophoretic injection of Neurobiotin (Vector Laboratories). Briefly, the tip of an intracellular recording electrode was filled with 5% Neurobiotin dissolved in 2 m KAc; the electrode was then backfilled with 2 m KAc and 20 mm KCl. Following cell impalement, we injected 2-nA negative current for a duration of 20 min. The ganglion was incubated at room temperature for 45 min following iontophoretic injection to allow the dye to diffuse to distal structures. Following this incubation period, the ganglion was fixed in 4% paraformaldehyde (overnight at 4°C) and rinsed in iso-osmotic Millonig’s buffer (all components from Sigma-Aldrich, recipe from Puhl et al., 2018). Cells were permeabilized in 1% Triton X-100 in iso-osmotic buffer for 2 h and incubated overnight at 4°C in a 1:50 dilution of streptavidin conjugated to Cy3 (Jackson ImmunoResearch). The ganglion was then rinsed in iso-osmotic Millonig’s buffer, dehydrated in ethanol, and mounted between glass coverslips using DEPEX mounting medium (VWR International). The filled Retzius cell was imaged on a Nikon A1 laser-scanning confocal microscope, and the resulting image was processed in ImageJ.

### Electrode displacement paradigm

For our electrode displacement paradigm (Fig. 1*E*), we rapidly raised and lowered the recording electrode by rotating the knob of our micromanipulator (Leitz joystick model, Leica Optical). Distance raised was tracked using marked notches on the fine-adjustment knob (each notch corresponds to a distance of 200 nm). The motion took ∼2 s, the fastest time in which we could consistently raise and lower the electrode. As with our US trials, electrode displacement was induced following a 20-s baseline recording, and subsequent trials had increased displacement until electrode impalement was lost.

### US characterization and parameters

**Figure 2.**
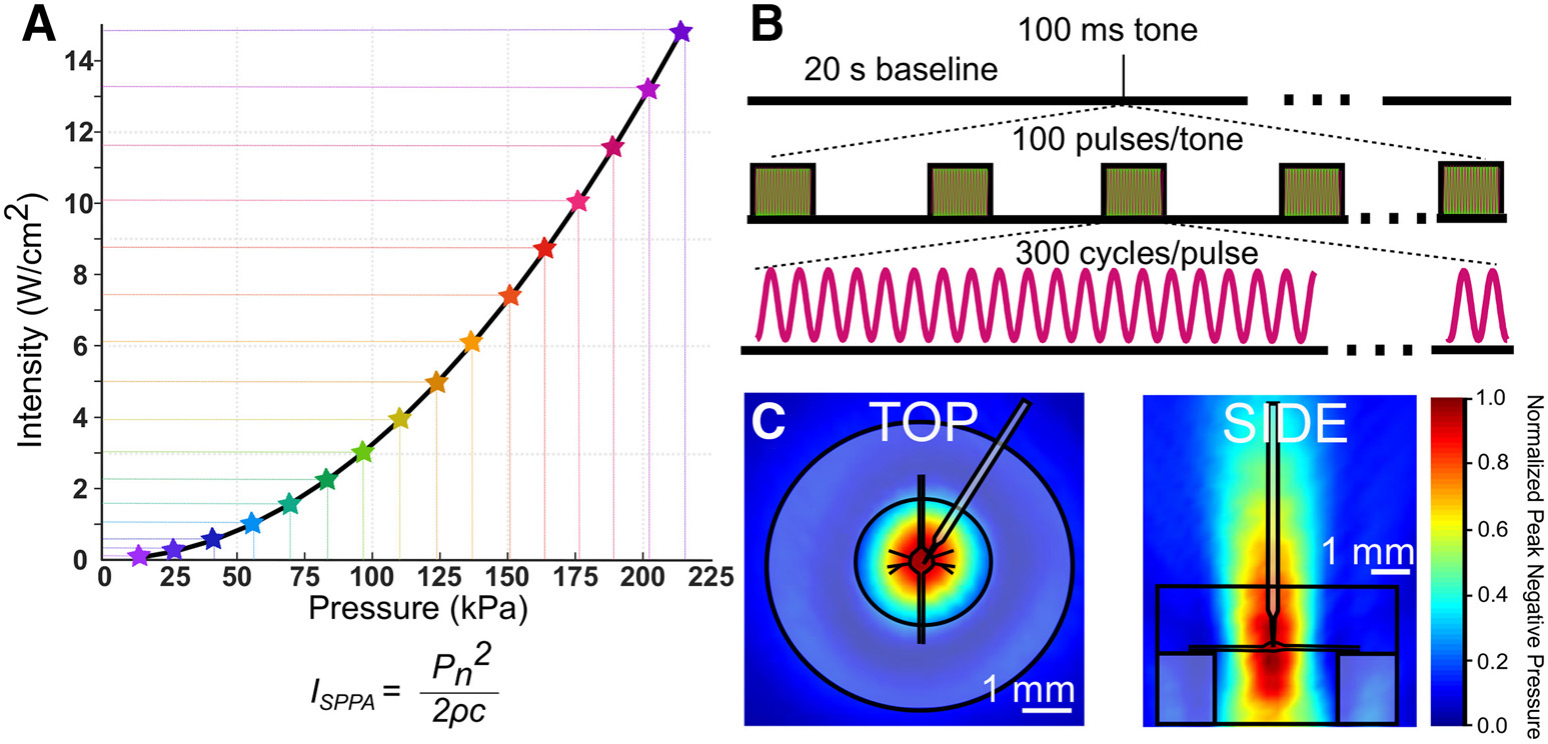
US parameters. ***A***, In this graph, all the pressures used in this study and their corresponding intensities (spatial peak pulse average) are indicated. Intensities were calculated using the equation shown in ***A***, where P_n_ = pressure; Þ = density of nerve tissue, estimated to be 1.03 × g/cm^3^; c = speed of sound in saline medium, estimated to be 1507 m/s. ***B***, US pulse parameters; 960-kHz US was applied for a single tone of 100-ms duration. Tones consisted of 100 pulses of 300 cycles of US (313-μs pulse duration). ***C***, Linearly interpolated pressure distribution maps overlaid with scale preparation, dish, and electrode.

All US waveforms were designed by a waveform generator (Agilent 33500B Series) and triggered by a TTL pulse from our intracellular recording digitizer via pClamp software. Waveforms were amplified by a 100-W RF linear power amplifier (E&I, model 2100L) and impedance matched with a matching network (Sonic Concepts). Transducer output was characterized by hydrophone (ONDA HNR-0500) measurements in 0.5-mm increments in *x*, *y*, and *z* directions in a large tank filled with deionized, degassed water. Shown in Figure 2*C* are the vertical and horizontal cross-sections of linearly interpolated hydrophone measurements (step size = 500 μm in *x*, *y*, and *z* directions; 309 total measurements) at peak amplitudes, which are overlaid with scaled preparation dimensions.

**Figure 3.**
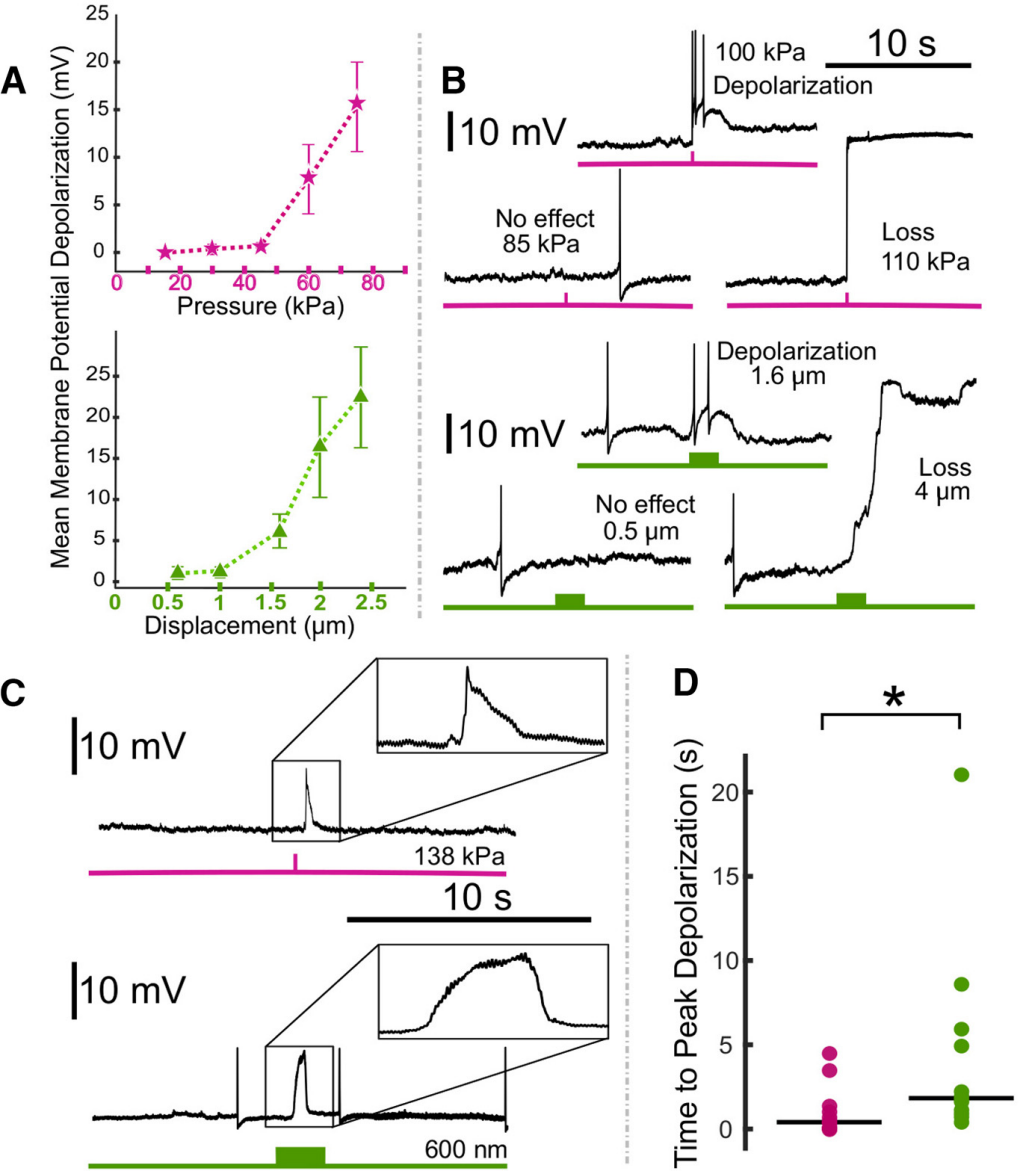
Comparison of the effects of US and electrode displacement on the resting membrane potential of Retzius neurons. ***A***, Plots demonstrating changes in mean membrane potential in response to US applied at increasing pressures (upper plot, pink) and electrode displacements of increasing distance (lower plot, green), aggregated across preparations. Error bars denote SEM. ***B***, Intracellular recordings demonstrating effects of US applied at increasing pressures to the same cell (pink, upper); recordings demonstrating effects of electrode displacement at increasing distances on the same cell (green, lower). ***C***, Intracellular recordings demonstrating typical waveforms of depolarizations elicited by US (upper) and electrode displacement (lower). ***D***, Scatter plots comparing time to peak depolarization following start of US (pink) and electrode displacement (green). Horizontal lines denote medians. The difference between the two was significant (*Z* = 2.6275, *p = 0.0086, Wilcoxon rank-sum test).

**Figure 4.**
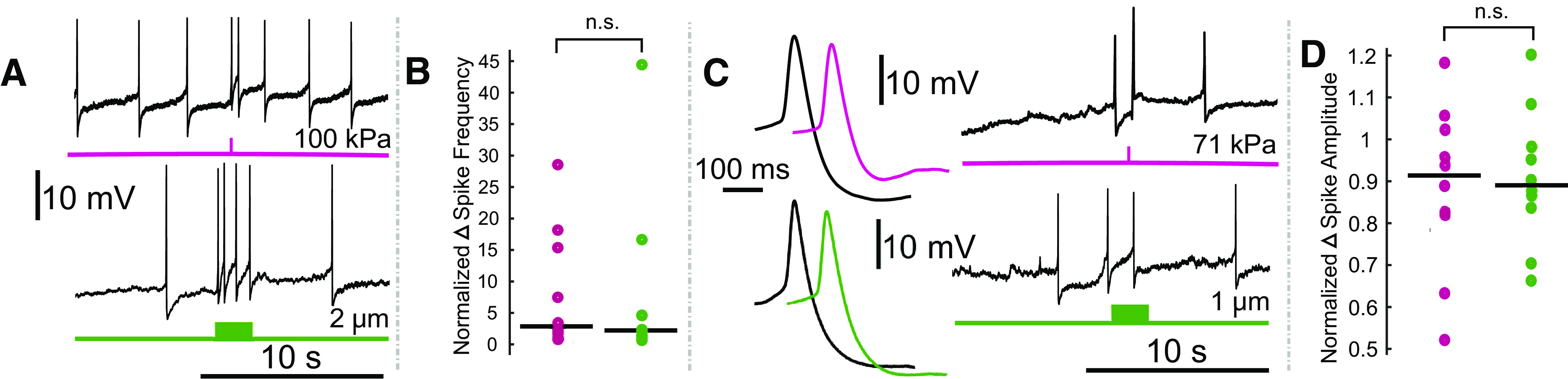
Comparison of the effects of electrode displacement on the spike frequency and amplitude of Retzius neurons. ***A***, Intracellular recordings demonstrating US (upper, pink) and electrode-displacement (lower, green) associated increase in spike frequency. ***B***, Scatter plots comparing the normalized change in spike frequency, during the period of peak effect, in US (pink) and electrode displacement (green) conditions. Horizontal lines denote medians. The difference between the two did not reach the threshold for significance (*Z* = 0.1890, *p* = 0.8501, Wilcoxon rank-sum test). ***C***, Intracellular recordings showing that US (pink) and electrode displacement (green) induce reductions in spike amplitude. Averaged spike waveforms (left) demonstrate reduction in spike amplitude (black waveforms = averaged from the two spikes before stimulus onset, pink and green waveforms = averaged from the two spikes fired during the peak effect period following US application and electrode displacement, respectively). ***D***, Scatter plots comparing normalized change in spike amplitude during peak effect period in US (pink) and electrode displacement (green) conditions. Horizontal lines denote medians. There was no significant (n.s.) difference between the two (*t*_(17.3329)_ = 0.2777, *p* = 0.7845, Welch’s *t* test).

In our first paradigm (Figs. 3, 4), US trials consisted of the application of a single tone of 960-kHz pulsed US for 100 ms following a 20-s baseline recording period. Pulses were 313 μs in duration and were delivered at a 1-kHz pulse repetition frequency. Peak pressures and intensities were increased sequentially in repeated trials until the electrode impalement was lost. Pulse parameters and the range of pressures and intensities used are described in Figure 2.

**Figure 5.**
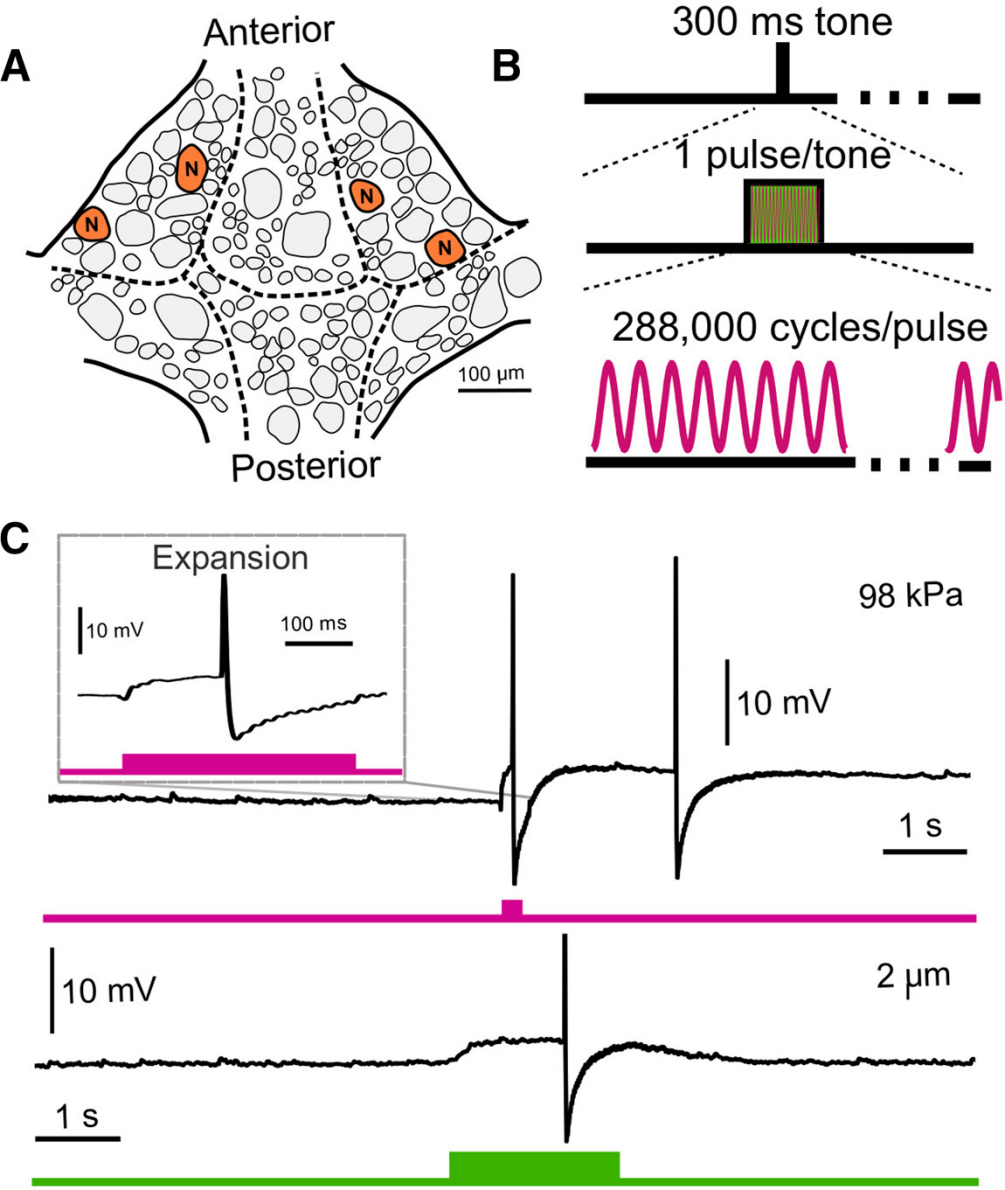
US application and electrode displacement yield similar results when a different neuron (N cell) and different pulse parameters are used. ***A***, Schematic of ventral surface of a single leech ganglion with N neurons marked. ***B***, US parameters applied to N cells. We applied one tone (300-ms duration) of continuous (vs pulsed) US per trial. ***C***, Representative intracellular traces of N cell voltage during a trial of US application (upper, pink) and electrode displacement (lower, green). When upper trace is expanded (inset), the waveform closely resembles that observed in the electrode displacement paradigm. The difference in the duration of the US-induced depolarization can be attributed to the difference in stimulus duration.

In our second paradigm (Fig. 5), US trials consisted of a single tone of 960-kHz continuous (100% duty cycle) US applied for 300 ms. Peak pressures and intensities were increased sequentially in repeated trials until electrode impalement was lost.

**Figure 6.**
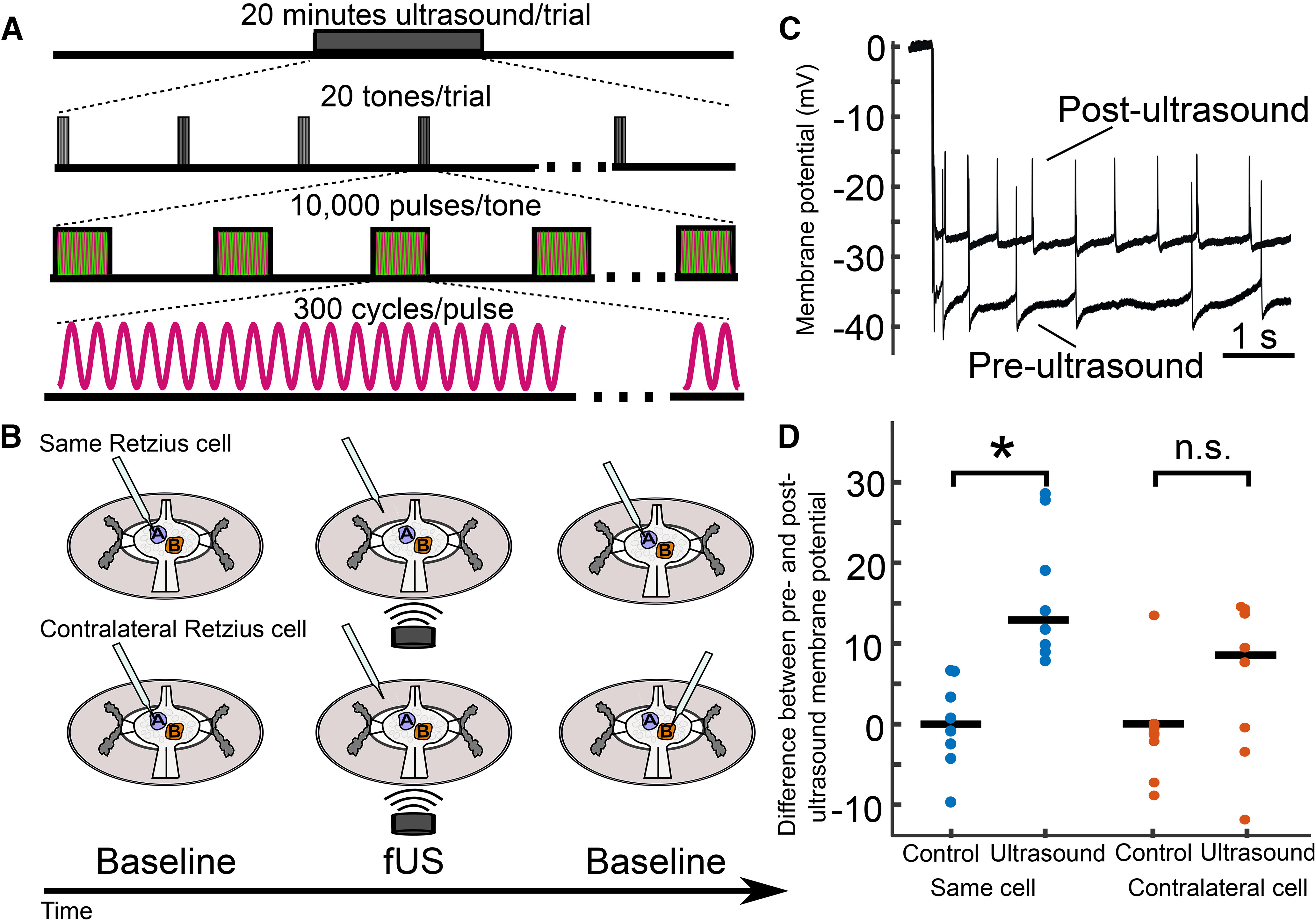
Retzius neuron membrane potential following extended US application is influenced by prior sharp electrode impalement. ***A***, Schematic of extended US application. Pulsed US was applied for a 20-min duration. Tones were delivered the first 10 s of each minute (tone duration = 10 s, tone frequency = 0.167 Hz). Tones consisted of 10,000 pulses of 300 cycles of 960-kHz US (pulse repetition frequency = 1 kHz, pulse duration = 312.5 μs). Pressure applied was 111 kPa in all trials. ***B***, Schematics of trial design for extended application paradigm. Upper, Retzius neuron was impaled (blue), and resting membrane potential was recorded. The recording electrode was then removed (middle cartoon) and US was applied for 20 min. Following US application, the electrode was re-inserted into the same Retzius cell for a second baseline recording. Lower, In a different preparation, the electrode was inserted into the Retzius cell (blue) to record the resting membrane potential. As in the previous experiment, the electrode was removed before 20 min of US application (middle cartoon). After application, the contralateral Retzius cell (orange) was impaled to record baseline activity; this cell was thus not previously impaled. ***C***, Intracellular recordings taken from the same Retzius cell before and after extended application of US demonstrating post-US depolarization of the resting membrane potential. ***D***, Scatter plots comparing differences between pre-US and post-US membrane potential (mV) in the same cell (blue) and contralateral cell (orange). Control paradigms replaced the US application period with a waiting period of equivalent time. Membrane potentials of the US-treated and control groups differed significantly (Wilcoxon rank-sum test, *p = 1.55e-4) when the same Retzius cell was re-impaled. However, the US and control groups showed no significant (n.s.) difference (Wilcoxon rank-sum test, *p* = 0.1605) when the contralateral cell was recorded.

In our third paradigm (Fig. 6), US trials consisted of a 20-min application of 960-kHz pulsed US preceded by a baseline recording period of at least 20 s. A subsequent baseline recording was made after the US application. US was applied for the first 10 s of every minute (tone duration = 10 s). Tones consisted of 313-μs pulses (pulse duration) pulsed at 1 kHz (pulse repetition frequency), yielding a duty cycle of ∼30%. US intensity and pressure were fixed at 4-W/cm^2^ spatial peak pulse average intensity (I_SPPA_) and 111 kPa, respectively.

### Statistics

All statistical tests save power analyses were performed in MATLAB. Data were tested for normality via Shapiro–Wilk tests. Comparisons of non-normally distributed data were performed via non-parametric Wilcoxon rank-sum tests; normally distributed data were compared via Welch’s *t* tests. All hypothesis tests were two-tailed with α = 0.05. We quantified effect sizes (Cohen’s *d* with correction for small sample sizes; Durlak, 2009) and performed *post hoc* power analyses. Power analyses were performed using G*Power 3.1 (Erdfelder et al., 2009). All statistical results are reported in Table 1.

**Table 1:** Descriptions of statistical tests

	Data structure	Type of test	Result	Effect size	Power
a	Non-normal US condition: *W*_(11)_ = 0.7185, *p* = 0.0018 ED condition: *W*_(11)_ = 0.6417, *p* = 4.38e-04	Wilcoxon rank-sum test	*Z* = 2.6275, *p* = 0.0086	*d* = 1.3018	0.8438
b	Non-normal US condition: *W*_(9)_ = 0.7890, *p* = 0.0141 ED condition: *W*_(9)_ = 0.5623, *p* = 2.6799e-04	Wilcoxon rank-sum test	*Z* = 0.1890, *p* = 0.8501	*d* = 0.0135	0.0501
c	Normal US condition: *W*_(9)_ = 0.9659 , *p* = 0.8508 ED condition: *W*_(9)_ = 0.9713 *p* = 0.9027	Welch’s *t* test	*t*_(17.3329)_ = 0.2777, *p* = 0.7845	*d* = 0.0343	0.0506
d	Non-normal US condition: *W*_(7)_ = 0.8499, *p* =0.0951 Control condition: W(7) = 0.9543 *p* = 0.7547	Wilcoxon rank-sum test	*Z* =100, *p* = 1.554E-4	*d* = 3.613	0.99
e	Non-normal US condition: *W*_(7)_ = 0.8802, *p* = 0.189 Control condition: *W*_(7)_ = 0.8802, *p* = 0.0274	Wilcoxon rank-sum test	*p* = 0.1605	*d* = 1.3432	0.68

Letters (leftmost column) correspond to *p* values of statistical tests as reported in Results. The data structure, test type, result, effect size, and statistical power of these tests are described. Results of Shapiro–Wilk test for normality of data in US and electrode displacement (ED) conditions (α = 0.05) are reported under Data structure. Normally distributed data were compared with Welch’s *t* test, and non-normal data were compared with the nonparametric Wilcoxon rank-sum test. Effect sizes were calculated as Cohen’s *d* with correction for small sample sizes as described by Durlak (2009).

## Results

### US depolarizes Retzius neurons and alters spike frequency and waveform

For the first set of experiments, depicted in Figures 3, 4, we applied US as described to 14 leech ganglia while recording intracellularly from one of the bilateral Retzius cells (*n* = 14 Retzius cells). Data from 2/14 recordings were not included in analyses because of an unstable baseline (membrane potential rising rapidly before US application because of poor electrode impalement); final *n* = 12. US induced a dose-dependent rise in the resting membrane potential, with higher pressures yielding greater depolarization. As US pressure increased in subsequent trials, neurons typically showed increasing levels of depolarization until the cell was lost, as evidenced by a sharp, high-amplitude increase in voltage consistent with partial or full loss of electrode impalement. Aggregated data demonstrating mean depolarization at ascending pressures are shown in Figure 3*A*; only data from the five lowest pressures are displayed, as these were sufficient to induce effects and/or loss in most of the cells tested, and thus our sample sizes at higher pressures were low. Responses were highly variable with respect to the pressures at which cells were lost (mean = 110.38 kPa, SD = 56.22). The mean time to peak depolarization following the US onset was 1.19 s (SD = 1.43). At maximally depolarizing pressures before loss (mean = 77.69 kPa, SD = 51.54), cells were depolarized by an average of 3.73 mV (SD = 3.25). We also observed changes in spike amplitude and spike frequency during peak depolarization (time from stimulus onset to beginning of a sustained period of repolarization toward baseline membrane potential). During peak depolarizations, most cells (*n* = 10/12) fired action potentials. Of these cells, mean spike amplitude (normalized to spike amplitude during 20-s baseline) was decreased (mean normalized spike amplitude = 0.88, SD = 0.20). Because changes in spike frequency were highly variable and the data were skewed, we have opted to report data dispersion versus mean and standard deviation. The median normalized spike frequency during the period of peak depolarization was 2.28; the interquartile range was 10.4. All data points are visible in Figure 4*B*.

Despite our awareness of others achieving similar results with respect to US-induced depolarization (Dedola et al., 2020), several factors gave us pause with respect to the legitimacy of our data. First, we observed high variability in responses to our tested pressures, which was less expected in this system than others because of our use of the same identified neuron in all preparations. Second, the sharp upward deflections in membrane potential even during moderate US-induced depolarizations were reminiscent of what we observed when a cell recording was naturally lost because of stochastic factors, a phenomenon that can occur in gradations (partial vs full loss), with a clear reduction in spike amplitude in instances in which partial electrode impalement remains. US causes mechanical disturbance of targeted tissue and can cause electrode resonance that can result in loss of contact with the recorded neuron (Tyler et al., 2008). We, like others, attributed cell loss resulting from US application to electrode resonance. We further suspected that US applications that fell below the pressure threshold to induce a full recording loss might induce a partial one, resulting in depolarization of the resting membrane potential and other reversible changes that, in isolation, could appear to be the cellular signatures of excitatory neuromodulatory processes.

### Electrode displacement mimics US-induced effects

To determine whether brief disruption of electrode placement could elicit effects comparable to US reliably, we performed trials in which we manually displaced the recording electrode in increasing increments while recording from Retzius cells in an additional 13 ganglia (*n* = 13 Retzius cells). The recording electrode was raised and lowered vertically in 2-s motions; displacement magnitude was standardized via notches on the micromanipulator knob corresponding to 200-nm distances. Data from one cell was not included in analyses because of an unstable baseline (final *n* = 12). Increasing displacements yielded dose-dependent depolarizations (Fig. 3*A*, means of data aggregated across cells). We observed high variability in the displacement magnitude necessary to lose cell impalement, with a mean of 3.93 μm (SD = 1.92). Time to maximum depolarization was also variable, occurring on average 4.34 s (SD = 5.83) from the start of the displacement motion. At maximally depolarizing displacements, before cell loss (mean = 2.38 μm, SD = 1.42), cells depolarized by an average of 3.62 mV (SD = 2.53). We also observed a reduction in spike amplitude and a reduction in spike frequency in the 10/12 cells that fired action potentials during the period of peak depolarization, similar to what we had observed with US. Mean normalized spike amplitude during peak US effects was 0.91 mV (SD = 0.16). Comparable to changes in spike frequency in the US condition, changes were highly variable and skewed, so we again opted to describe data dispersion versus mean and standard deviation. The median normalized spike frequency during the period of peak depolarization was 2.24; the interquartile range was 3.23. All data points are visible in Figure 4*B*.

Both US and manual electrode displacement were found to depolarize cells up to a threshold that resulted in a loss of the intracellular recording; examples may be seen in Figure 3*B*, in which traces show typical outcomes in a cell exposed to US at increasing pressures (Fig. 3*B*, upper, pink), and a cell subjected to electrode displacement (Fig. 3*B*, lower, green). Time to peak depolarization differed between the two conditions (Fig. 3*C*,*D*); *Z* = 2.6275, *p* = 0.0086^a^. This difference is consistent with the differential in stimulus application time (100 ms for US vs 2 s for electrode displacement). We observed an increase in spike frequency and a decrease in spike amplitude in both US and electrode displacement conditions (Fig. 4*A–D*). Mean increase in spike frequency and decrease in spike amplitude at maximally depolarizing levels before loss did not differ significantly between US and electrode displacement (spike frequency: *Z* = 0.1890, *p* = 0.8501^b^, Wilcoxon rank-sum test; spike amplitude: *t*_(17.33)_ = 0.2777, *p* = 0.7843^c^, Welch’s *t* test).

### The depolarizing effects of US and electrode displacement are common to N neurons

To assess whether our observed effects were applicable to other identified neurons in the leech, we performed an additional set of experiments on another cell type, the N cell (Fig. 5*A*). This cell was chosen because of its usage in a recent study in which US was reported to depolarize leech neurons in an intracellular paradigm (Dedola et al., 2020). We adjusted pulse parameters to mimic more closely those found to be effective in eliciting a response in N cells: we applied a single pulse of continuous US with a 300-ms pulse duration (Fig. 5*B*). We were unable to replicate fully the authors’ paradigm as we were constrained by the higher center frequency of our US transducer (960 vs 490 kHz).

We applied US at ascending pressures to six N cells (*n* = 6) while recording intracellularly. Our first tested pressure was 20 kPa (root mean squared, the highest pressure used by Dedola et al., 2020); we observed that 0/6 cells responded. Increasing pressures, however, were sufficient to elicit depolarization and, ultimately, loss of electrode impalement. At maximally depolarizing pressures before recording loss (mean = 49.3 kPa, SD = 30.5), mean depolarization was 3.50 mV (SD = 4.11). A representative trace of this depolarization is displayed in Figure 5*C*, upper.

We next assessed whether these effects could be mimicked by electrode displacement in a manner comparable to what we observed in Retzius cells. We again displaced the recording electrodes by ascending distances until the intracellular recording was lost. We observed a similar phenomenon, in which electrode deflections insufficient to compromise the recording resulted in small depolarizations. Maximal depolarization before loss of electrode impalement was achieved at 2.25 μm (SD = 0.99) and averaged 3.45 mV (SD = 3.45). A representative trace of this effect is displayed in Figure 5*C*, lower.

### US application following electrode impalement depolarizes Retzius neurons

Our results in both cell types raised concern as to whether US-induced changes in the resting membrane potential of neurons could be accurately assessed via intracellular recording during US application. We next sought to determine whether it was feasible to measure changes by comparing baseline characteristics from the same cell before and after US application. The large, physiologically robust, and easily identifiable nature of the Retzius neurons enabled reentry into the same cell in 20–30 s following cessation of US application. We were concerned that the effects of a 100 ms application of pulsed US, as we had used in our previous experiment, would not persist for the time taken to re-enter the cell. Assuming longer application times yielded more persistent effects, we dramatically increased the US application period to 20 min. US parameters for these experiments are outlined in Figure 6*A*; the broader experimental design is outlined in Figure 6*B*.

We found that Retzius neurons (*n* = 8) exposed to 20 min of US were depolarized from their pre-US baseline (mean change = 16.03 mV, SD = 8.29). Neurons re-entered after a 20-min wait period with no US (control condition, *n* = 8) did not have a demonstrable change in membrane potential (mean change = 0.0625 mV, SD = 5.57). The change in membrane potential in the US versus control conditions differed significantly (*Z* = 100, *p* = 1.554E-4^d^, Wilcoxon rank-sum test). Intracellular traces recorded in the same cell before and after US application are shown for comparison in Figure 6*C*.

Despite this compelling result, we were concerned that the depolarization we observed as a function of US application could still have resulted from electrode-associated artifactual effects, including creation of a leaking puncture in the cell membrane, or the introduction of cavitational nuclei. As a control, we performed a similar experiment in which we recorded from the contralateral Retzius neuron following US application instead of the same cell (Fig. 6*B*, lower, schematic). The two Retzius neurons in each ganglion are electrically coupled and are known to be isopotential (Hagiwara and Morita, 1962; Eckert, 1963). Recording from the contralateral cell yielded an opportunity to estimate changes in membrane potential caused by US in an electrode-naive cell. Intriguingly, the depolarization we observed in the same-cell condition did not persist significantly in the contralateral condition (*p* = 0.1605, Wilcoxon rank-sum test), suggesting the stark depolarization we observed in the same-cell condition could have been influenced by the initial electrode impalement.

## Discussion

### Overview

We have demonstrated that US reliably produces a dose-dependent depolarization of the resting membrane potential of single leech Retzius neurons when applied during intracellular sharp-electrode recording. We found that these effects, however, are likely to be artifactual as they could be mimicked by the manual displacement of the recording electrode. US effects appeared to differ from manual electrode displacement only with respect to the time to achieve peak effects. We believe that this difference is simply because of the time course of the applied stimulus across the two paradigms; for example, US was delivered for 100 ms, while manual displacement and replacement of the electrode took longer (∼2 s). We also determined that even when the recording electrode was removed from the targeted neuron during US application, the baseline (i.e., first) impalement appeared to cause a sufficient leak current to affect the subsequent membrane properties of the Retzius cell when recorded after US application (Fig. 6). In contrast, by recording from the electrode-naive contralateral Retzius neuron, which was impaled only once and after the US was applied, we observed that US did not induce a statistically significant elevation in resting membrane potential.

We observed similar results, as discussed above, when targeting N cells, sensory neurons recently reported to depolarize during US application (Dedola et al., 2020). Using one of the authors’ employed pulse parameters (300 ms of continuous US), we observed depolarization of a comparable magnitude. Achieving this effect, however, required the use of higher pressures than the authors reported, which we attribute to our use of a higher US frequency. Higher frequencies (with shorter wavelengths) generate less electrode resonance. As we suspect that electrode resonance is a primary driver of depolarization in intracellular paradigms, it follows that higher pressures may be required to elicit comparable depolarizations when working with higher US frequencies. Importantly, by briefly displacing the recording electrode, we were able to mimic the effects of US on the N cells as well.

We conclude that a nonspecific leak current most likely contributes to the US-induced depolarizations we observed. In leech neurons, it has been shown previously that sharp electrode impalement can affect nonspecific leak currents, having profound effects on the ability of some cells, for example, to exhibit endogenous bursting activity (Cymbalyuk et al., 2002).

### The confounds of electrode recording techniques

US-induced electrode resonance is a commonly-reported problem, complicating efforts to asses US effects via whole-cell patch clamp (Tyler et al., 2008; Prieto et al., 2018) and two-electrode voltage clamp (Kubanek et al., 2016). Although these reports used different single-cell recording modalities, some of the electrophysiological signatures of neuromodulation following US onset resemble our own, characterized by a very steep initial depolarization that elicits action potentials (Tyler et al., 2008). This steep depolarization and increase in spike frequency were observed similarly in a recent intracellular sharp electrode study of the actions of US on a type of leech sensory neuron (Dedola et al., 2020). These authors also reported a US-associated reduction in spike amplitude, which is consistent with our US and electrode displacement data. We cannot rule out the possibility that US can induce a rapid depolarization, at certain US parameters and in some types of neurons across animal models, as suggested by prior work using optophysiological techniques (Tyler et al., 2008; Qiu et al., 2019). We can, however, strongly posit that electrode resonance is a potent indirect driver of US-induced neuronal stimulation in the context of intracellular paradigms, especially in the leech.

Concerns of artifactual effects have been raised previously, when it was postulated that US-induced electrode resonance, particularly at sub-MHz frequencies, could introduce depolarizing leak currents in *Xenopus* oocytes (Kubanek et al., 2016). It remains unclear whether extracellular recordings are similarly prone to artifactual effects when combined with US. Minute movements of an animal preparation or displacement of any type of electrode induced by US could cause a temporary reduction in electrode resistance, yielding an artifactual reduction in voltage as measured, for example, in the form of a reduced-amplitude single or compound action potential.

One additional concern in combining US with single-cell electrophysiological recording techniques is the potential to introduce cavitational nuclei. US has been theorized to depolarize neurons through the rhythmic expansion and contraction of microbubbles in the cell membrane, altering membrane capacitance (Krasovitski et al., 2011; Plaksin et al., 2014). Electrode insertion could transport non-endogenous cavitational nuclei to the cell membrane from the surrounding media, facilitating US effects. Degassing the saline medium, as was done in our report, may limit the potential for artifactual cavitational effects. However, aerating bath disturbances caused by insertion and movement of the recording electrode remain potential considerations. The introduction of cavitational nuclei may be of particular concern with mammalian preparations that require continued oxygenation.

### Alternative approaches

Moving forward, reducing the confounds of electrode resonance will be important to achieve confidence in defining the cellular underpinnings of US’s actions. Resonance can be reduced by separating the recording site from the site of US application (e.g., applying US to a neuron’s axon while recording from the soma). This is an imperfect solution, however, as distal changes to membrane properties may not be accurately reflected at the soma because of space clamp issues (Spruston and Johnston, 2008). Another potential means of reducing resonance is by increasing US frequency, thereby decreasing wavelength, a strategy with which other groups have found success (Prieto et al., 2018; Ye et al., 2018). Although this latter strategy may be effective in reducing resonance, it cannot eliminate it entirely, and there remains the potential for a resonating electrode to cause a leak at the site of electrode entry, increasing cell permeability to surrounding sodium-rich media and inducing artifactual depolarization. In addition, it remains unclear whether US at frequencies in the 10 s of MHz range, as used in these studies, affect neural function in a manner comparable to US in the 100 s of kHz range used in transcranial studies (Tufail et al., 2010; Min et al., 2011; Legon et al., 2014, 2018; Lee et al., 2016).

In conclusion, we are of the opinion that future investigations exploring the effects of US on single neurons should avoid simultaneous intracellular recording and US delivery. Investigations that incorporate extracellular or optical recording approaches may be better suited to control for the potential artifactual effects of electrode resonance, an idea already adopted by some other groups who have found success with optical alternatives to classical electrophysiological techniques, including the use of ion-indicator dyes (Tyler et al., 2008; Qiu et al., 2019).

## Synthesis

Reviewing Editor: Elizabeth Cropper, Mt Sinai Sch Med Decisions are customarily a result of the Reviewing Editor and the peer reviewers coming together and discussing their recommendations until a consensus is reached. When revisions are invited, a fact-based synthesis statement explaining their decision and outlining what is needed to prepare a revision will be listed below. The following reviewer(s) agreed to reveal their identity: Ronald Calabrese, Hillel Chiel.

As is described in more detail below the reviewers felt that this is an interesting paper that addresses a potentially important problem, data presented are consistent with the proposed hypothesis, and experiments are carefully done. There were however two major concerns. One concerned the discussion of unpublished results in the Introduction. The suggestion is to either remove this material or add some of these data to the current manuscript. Adding data would strengthen the arguments made in this report. Secondly, both reviewers had issues with the fact that data in the present manuscript were not obtained using the neurons and techniques of the study that is being refuted (Dedola et al.) (as is described in more detail in Review #2). The reviewers request that the authors either discuss this point or modify their conclusions concerning the generality of their findings.

Detailed reviews:

Reviewer #1

The authors use sharp microelectrode electrophysiology combined with quantified application of focused ultrasound fUS to test the hypothesis that depolarization induced by fUS in leech neurons is due to electrode displacement due to resonance. They were led to these experiments by unpublished work from their lab showing suppression of activity in similar leech neurons recorded extracellularly. The data that they present is consistent with their hypothesis. The experiments appear carefully done and the data robust.

Major Concerns

1. I think it is unusual to describe unpublished results in the Introduction and then refer to them again in Discussion as manuscript submitted “Finally, we are confident that the effects of fUS, which are not artifactual (manuscript submitted), indicate that fUS induces neuronal inhibition and action potential conduction block...” The authors should wait till that manuscript is published (accepted for publication and available for evaluation) before submitting this one or drop reference to the work. There is not even a bioRxiv link to the work.

Minor Concerns

1. The writing in Results is not always precise. It is not always clear what the stimulus amplitude (of the fUS) is for each record shown (In Figure 3b, an amplitude series is given but there is no indication of fUS amplitude for each trace.) In Figure 5 experiment it is not clear whether the same stimulus amplitude is used throughout OR whether this at maximally depolarizing levels prior to loss. It is not clear whether the descriptive statistics are shown with sd or sem. The authors refer to lack of electrode contact often, when I think them mean impalement of penetration. The electrode probably remains in contact with the cell just not penetrating the membrane.

2. Data Presentation. The statistical Table 1 require a more extensive description in Methods or legend for this reviewer to fully understand it. Descriptive statistics sometimes seem absurd such as on line 244: “Mean normalized spike frequency was 7.93 {plus minus} 13.67...” Because spike frequency cannot be negative this sd (is it an sd or an sem?) indicates tremendous skew and lack of normality. Is it valid to give sd under these conditions?

3. Would electrode buzz (overcompensation of electrode capacitance) be a good mimic of ultrasound induced electrode resonance?

Reviewer #2

This is an interesting paper that addresses a potentially important problem, which is determining the role of focused ultrasound in controlling neural activity.

The major focus of the paper is to argue that the effects of focused ultrasound that have been previously studied in neurons within the nervous system of the leech, which led to the conclusion that focused ultrasound induces excitation (Dedola et al. 2020) is essentially an artifact caused by the movements that ultrasound induces in a microelectrode that is impaling the neurons.

The authors claim that the real effect of focused ultrasound is exactly the opposite: “Recently, we obtained evidence to support the idea that the direct effects of fUS are largely thermal in nature, resulting in the suppression of neuronal firing....Because we used extracellular suction electrodes versus intracellular or patch electrodes to record action potentials from the axons of our identified neuron, we considered whether different recording methodologies might contribute to a phenomenon of excitation versus inhibition.” (text starting on page 4, line 68).

The authors reiterate this important claim near the end of the paper: “Finally, we are confident that the effects of fUS, which are not artifactual (manuscript submitted), indicate that fUS induces neuronal inhibition and action potential conduction block, accompanied by a stimulus-to-response profile that greatly differs from the rapid depolarizing responses obtained in the study here and that of Dedola et al. (2020).” (text starting on page 16, line 329).

Thus, the goal of this paper is to argue that the results of Dedola et al.(2020) and others that have reported excitatory effects of focused ultrasound are essentially all artifactual.

This is an important claim, and the data shown by the authors does suggest that mechanical disturbances to an electrode can generate depolarizations similar to those generated by focused ultrasound.

I have two major concerns with the paper.

(1) In Dedola et al., the following protocol was used for ultrasound: “Sinusoidal tone bursts at 490 kHz, with pressure amplitude from 8 to 20 kPa were delivered at a pulse repetition frequency (PRF = 1/PRP) of 10 Hz, for a total stimulus duration of 300 ms.” (p. 31); Dedola et al. looked at the effect of varying the duty cycle; they also put considerable effort into characterizing the ultrasound field as precisely as possible. Finally, Dedola et al. used Nociceptive (N) and Pressure (P) neurons in the leech for their study.

In the current study, the authors used different ultrasound parameters: “fUS trials consisted of application of a single tone of 960 kHz pulsed ultrasound for 100 ms following a 20 second baseline recording period. Pulses were 312.5 microseconds in duration and were delivered at a 1 kHz pulse repetition frequency.” (p. 8, line 158). In the current study, the authors used Retzius neurons.

The authors claim that their results are essentially identical to those of Dedola et al.: “This steep depolarization and increase in spike frequency were observed similarly in a recent intracellular sharp electrode study of the actions of fUS on a type of leech sensory neuron (Dedola et al., 2020). These authors also reported an fUS-associated reduction in spike amplitude, which is consistent with our fUS and electrode displacement data.” (p.16, line 323).

How can the authors be confident that their results completely negate the results of Dedola et al. if they did not use the same ultrasound parameters, and the same neurons as Dedola did? The authors should provide a justification for using different parameters and different neurons, and then claiming that the results of these different parameters and different neurons should cause identical effects, especially since Dedola et al. reported somewhat different responses in N versus P neurons. If they do not, then their observations are certainly of interest, but do not necessarily negate the results of Dedola et al.

(2) The authors have decided to submit a separate manuscript describing what they argue are the “true” effects of focused ultrasound, which (as quoted above) are inhibitory. What is the reason that they did not report some of this data in the manuscript under consideration as a positive control for their argument that the effects of ultrasound when measured using sharp micro electrodes is all artifactual? This would provide far more convincing evidence for their contention that the only depolarizing effects of focused ultrasound are through the micro electrodes.

## Author Response

We would like to thank the reviewers for their thoughtful and constructive comments. This feedback enabled us to recognize areas in which our paper could be improved, and we have done our best to incorporate all reviewers’ suggestions. Both reviewers were concerned by our inclusion of a reference to unpublished data, which we have rectified by replacing this reference with a published source. We also softened our language to prevent implication of claims unsupported by the data in the present study. In response to a Major Concern posed by Reviewer 2, we performed additional experiments to better align our results with those of a study that we reference frequently. We sincerely hope that we have adequately addressed both reviewers’ concerns, and trust that incorporation of their comments has strengthened this manuscript to its current form.

REVIEWER 1

Major Concerns

Reviewer 1:

I think it is unusual to describe unpublished results in the Introduction and then refer to them again in Discussion as manuscript submitted “Finally, we are confident that the effects of fUS, which are not artifactual (manuscript submitted), indicate that fUS induces neuronal inhibition and action potential conduction block...” The authors should wait till that manuscript is published (accepted for publication and available for evaluation) before submitting this one or drop reference to the work. There is not even a bioRxiv link to the work.

Authors’ response:

We apologize for including unpublished results without proper citations. In part, we were unsure of how to include such information without compromising anonymity. The major results we wanted to reference were described in a manuscript that was in review at the time of submission of this manuscript; our hope was for this other manuscript to be published in time to include it as a reference in the current manuscript. In its place, we have cited supporting work contained in a refereed book chapter by the authors (lines 66-67), which includes electrophysiological traces demonstrating our mentioned inhibitory effects of US. In addition, we cite the authors who are on the paper that is in revision. We also removed all language above, as suggested, and any reference to US causing action potential conduction block, which is not fully described in our book chapter.

Minor Concerns

Reviewer 1:

1. The writing in Results is not always precise. It is not always clear what the stimulus amplitude (of the fUS) is for each record shown (In Figure 3b, an amplitude series is given but there is no indication of fUS amplitude for each trace.)

Authors’ response:

We agree that failing to include the amplitudes used to generate the depicted results was an oversight, which we have remedied.

Reviewer 1:

In Figure 5 experiment it is not clear whether the same stimulus amplitude is used throughout OR whether this at maximally depolarizing levels prior to loss.

Authors’ response:

The stimulus amplitude for these experiments is described as fixed at 111 kPa in the Methods (lines 199-200). We acknowledge that this is quite easy to miss, so we have made an additional note in the figure caption (Fig. 6, lines 627-628).

Reviewer 1:

It is not clear whether the descriptive statistics are shown with sd or sem.

Authors’ response:

Values were reported as “mean ± SD.” This was briefly mentioned in parentheses in Results, but it was easy to miss. To increase transparency, we replaced this reporting style with “mean = X, SD = Y.”

Reviewer 1:

The authors refer to lack of electrode contact often, when I think them mean impalement of penetration. The electrode probably remains in contact with the cell just not penetrating the membrane.

Authors’ response:

We agree that describing this phenomenon as a loss of membrane penetration provides a more accurate description of our presumed effect, and we have thus revised our wording to refer to a loss of impalement.

Reviewer 1:

2. Data Presentation. The statistical Table 1 require a more extensive description in Methods or legend for this reviewer to fully understand it.

Authors’ response:

We agree that Table 1 needed clarification. We have expanded the table caption (lines 647-655):

"Letters (leftmost column) correspond to p-values of statistical tests as reported in Results. The data structure, test type, result, effect size, and statistical power of these tests are described. Results of Shapiro-Wilk test for normality of data in ultrasound (US) and electrode displacement (ED) conditions (α=0.05) are reported under “Data Structure.” Normally distributed data were compared with Welch’s t test, and non-normal data were compared with the nonparametric Wilcoxon rank-sum test. Effect sizes were calculated as Cohen’s d with correction for small sample sizes as described by Durlak (2009).”

Reviewer 1:

Descriptive statistics sometimes seem absurd such as on line 244: “Mean normalized spike frequency was 7.93 {plus minus} 13.67...” Because spike frequency cannot be negative this sd (is it an sd or an sem?) indicates tremendous skew and lack of normality. Is it valid to give sd under these conditions?

Authors’ response:

We have clarified that values are reported alongside SD by changing wording to “mean = X, SD = Y.” This also avoids confusing readers by suggesting the possibility of negative values for metrics such as spike frequency.

With respect to the skew and lack of normality of our spike frequency data, we agree that these data are highly variable. All data points used to calculate the mean and SD are represented graphically in Fig. 4b to guide readers curious about our data distribution. We are unaware of situations in which it is invalid to report standard deviation, even in cases of high variability.

Reviewer 1:

3. Would electrode buzz (overcompensation of electrode capacitance) be a good mimic of ultrasound induced electrode resonance?

Authors’ response:

This is a thoughtful suggestion. We did initially explore whether electrode buzzing could be used to mimic ultrasound, as it generates electrode resonance. Unfortunately, the rapid overcompensation of capacitance caused high-amplitude “ringing” artifact in our voltage recording for the duration of the buzz. It was thus difficult to determine the magnitude of buzz-induced changes in membrane potential, or observe immediate changes in spike firing rate. We thus chose to pursue manual displacement, which, though slower, did not generate artifact, and had the additional advantage of measurability of electrode displacement distance by quantifiable increments.

REVIEWER 2

Major concerns

Reviewer 2:

(1) In Dedola et al., the following protocol was used for ultrasound: “Sinusoidal tone bursts at 490 kHz, with pressure amplitude from 8 to 20 kPa were delivered at a pulse repetition frequency (PRF = 1/PRP) of 10 Hz, for a total stimulus duration of 300 ms.” (p. 31); Dedola et al. looked at the effect of varying the duty cycle; they also put considerable effort into characterizing the ultrasound field as precisely as possible. Finally, Dedola et al. used Nociceptive (N) and Pressure (P) neurons in the leech for their study.

In the current study, the authors used different ultrasound parameters: “fUS trials consisted of application of a single tone of 960 kHz pulsed ultrasound for 100 ms following a 20 second baseline recording period. Pulses were 312.5 microseconds in duration and were delivered at a 1 kHz pulse repetition frequency.” (p. 8, line 158). In the current study, the authors used Retzius neurons.

The authors claim that their results are essentially identical to those of Dedola et al.: “This steep depolarization and increase in spike frequency were observed similarly in a recent intracellular sharp electrode study of the actions of fUS on a type of leech sensory neuron (Dedola et al., 2020). These authors also reported an fUS-associated reduction in spike amplitude, which is consistent with our fUS and electrode displacement data.” (p.16, line 323).

How can the authors be confident that their results completely negate the results of Dedola et al. if they did not use the same ultrasound parameters, and the same neurons as Dedola did? The authors should provide a justification for using different parameters and different neurons, and then claiming that the results of these different parameters and different neurons should cause identical effects, especially since Dedola et al. reported somewhat different responses in N versus P neurons. If they do not, then their observations are certainly of interest, but do not necessarily negate the results of Dedola et al.

Authors’ response:

This is an important point. While our intention was to provide a broad cautionary tale about the artifactual effects of electrode resonance, a secondary goal was to generate results comparable to those of Dedola et al.

To that end, we performed additional experiments. We chose to target the N cell, as it was reported to be more responsive to ultrasound than the P cell, and adjusted our pulse parameters to mimic those of Dedola et al. Specifically, we used 300 ms pulses (the authors’ longest pulse duration) of continuous ultrasound (the authors’ highest duty cycle). We were not able to replicate their lower frequency, as commercial ultrasound transducers such as ours operate within a fairly narrow range of frequencies.

We were able to depolarize N cells with application of ultrasound at these parameters, albeit at a slightly higher pressure than that reported by Dedola et al., which we attribute to our use of a higher frequency, which generates less electrode resonance when all other factors are equal. Importantly, similar to what we observed with Retzius cells, we were able to replicate ultrasound-associated depolarization by displacing the recording electrode. These data are described in a new section in the Methods (lines 190-192), Results (“The depolarizing effects of ultrasound and electrode displacement are common to nociceptive neurons"; lines 291-316) and shown in the new Figure 5 (Caption: lines 624-647). They are also summarized in the Discussion (lines 371-382).

Reviewer 2:

(2) The authors have decided to submit a separate manuscript describing what they argue are the “true” effects of focused ultrasound, which (as quoted above) are inhibitory. What is the reason that they did not report some of this data in the manuscript under consideration as a positive control for their argument that the effects of ultrasound when measured using sharp micro electrodes is all artifactual? This would provide far more convincing evidence for their contention that the only depolarizing effects of focused ultrasound are through the micro electrodes.

Authors’ response:

This was also a concern of Reviewer 1. We did not include those data in this manuscript as we (a) intend to report them in a comprehensive study to be published separately, and (b) wanted this manuscript to focus on the potential for artifact to obscure ultrasound’s effects versus the effects themselves. Those data were collected using a fairly different paradigm (extracellular vs. intracellular recording, long application versus short application, targeting the axon of a motoneuron versus the soma of an interneuron). It would thus be difficult to include them while maintaining focus on the current experiments. We have opted instead to modify our description of these data.

We also realize that our language implied that we believed that ultrasound’s only possible depolarizing effects are due to electrode resonance, which was not our intention, and is a bolder claim than our data can support. Ultrasound’s parameter space is vast, and it is possible that certain applications are able to depolarize neurons. We thus softened our wording in several places to clarify our point, which is that electrode resonance obscures effects, rather than it being the sole possible driver of ultrasound depolarization. Both sections below have been lightly reworded to specify that our results pertain to single cell recording paradigms, and are not intended to disprove potential depolarizing effects of ultrasound.

In the introduction (lines 105-108):

"Due to known US-induced electrode resonance, the rapid depolarization of cells found to occur in neurons in response to US application during intracellular recording may be artifactual...”

In the discussion (lines 404-408):

"Although we cannot rule out the possibility that US can induce a rapid depolarization, at certain US parameters and in some types of neurons across animal models, we can strongly posit that electrode resonance is a potent indirect driver of US-induced neuronal stimulation in the context of intracellular paradigms, especially in the leech.”

ADDITIONAL EDITS

Nomenclature

Based on feedback we have received from others in the ultrasound community, we have opted to replace our focused ultrasound acronym “fUS” with the more common “US", as we were advised that “fUS” can refer to functional ultrasound.

Methods

We included a new section on hydrophone characterization of our ultrasound transducer. It was our original intent to reference this characterization described in depth in a separate manuscript, but it appears this manuscript will be published first, and thus we shifted the description to this paper. This may also help to assuage a concern of Reviewer 2, which is that our ultrasound field was less well-characterized than that of Dedola et al.

New text, from lines 168-177:

"All US waveforms were designed by a waveform generator (Agilent 33500B Series) and triggered by a TTL pulse from our intracellular recording digitizer via pClamp software. Waveforms were amplified by a 100 W RF linear power amplifier (E&I, model 2100L) and impedance matched with a matching network (Sonic Concepts). Transducer output was characterized by hydrophone (ONDA HNR-0500) measurements in 0.5 mm increments in x, y, and z directions in a large tank filled with deionized, degassed water. Shown in Fig. 2c are the vertical and horizontal cross-sections of linearly interpolated hydrophone measurements (step size = 500 microns in x, y, and z directions; 309 total measurements) at peak amplitudes, which are overlaid with scaled preparation dimensions.”
